# Polymorphisms in Fatty Acid Desaturase (*FADS*) Gene Cluster: Effects on Glycemic Controls Following an Omega-3 Polyunsaturated Fatty Acids (PUFA) Supplementation

**DOI:** 10.3390/genes4030485

**Published:** 2013-09-10

**Authors:** Hubert Cormier, Iwona Rudkowska, Elisabeth Thifault, Simone Lemieux, Patrick Couture, Marie-Claude Vohl

**Affiliations:** 1 Institute of Nutrition and Functional Foods (INAF), Laval University, Quebec City, QC, G1V 0A6, Canada; E-Mails: hubert.cormier.1@ulaval.ca (H.C.); iwona.rudkowska@fsaa.ulaval.ca (I.R.); elisabeth.thifault@fsaa.ulaval.ca (E.T.); simone.lemieux@fsaa.ulaval.ca (S.L.); patrick.couture@crchul.ulaval.ca (P.C.); 2 Endocrinology and Nephrology, Laval University Medical Research Center, Quebec City, QC, G1V 0A6, Canada; 3 Lipid Research Center, CHUL Research Center, Quebec City, QC, G1V 4G2, Canada

**Keywords:** metabolic pathways, genotype, *FADS* gene cluster, polyunsaturated fatty acid omega-3, insulin insensitivity, glucose metabolism, homeostasis model assessment

## Abstract

Changes in desaturase activity are associated with insulin sensitivity and may be associated with type 2 diabetes mellitus (T2DM). Polymorphisms (SNPs) in the fatty acid desaturase (*FADS*) gene cluster have been associated with the homeostasis model assessment of insulin sensitivity (HOMA-IS) and serum fatty acid composition. **Objective:** To investigate whether common genetic variations in the *FADS* gene cluster influence fasting glucose (FG) and fasting insulin (FI) responses following a 6-week *n*-3 polyunsaturated fatty acids (PUFA) supplementation. **Methods:** 210 subjects completed a 2-week run-in period followed by a 6-week supplementation with 5 g/d of fish oil (providing 1.9 g–2.2 g of EPA + 1.1 g of DHA). Genotyping of 18 SNPs of the *FADS* gene cluster covering 90% of all common genetic variations (minor allele frequency ≥ 0.03) was performed. **Results:** Carriers of the minor allele for rs482548 (*FADS2*) had increased plasma FG levels after the *n*-3 PUFA supplementation in a model adjusted for FG levels at baseline, age, sex, and BMI. A significant genotype*supplementation interaction effect on FG levels was observed for rs482548 (*p* = 0.008). For FI levels, a genotype effect was observed with one SNP (rs174456). For HOMA-IS, several genotype*supplementation interaction effects were observed for rs7394871, rs174602, rs174570, rs7482316 and rs482548 (*p* = 0.03, *p* = 0.01, *p* = 0.03, *p* = 0.05 and *p* = 0.07; respectively). **Conclusion:** Results suggest that SNPs in the *FADS* gene cluster may modulate plasma FG, FI and HOMA-IS levels in response to *n*-3 PUFA supplementation.

## 1. Introduction

Type 2 diabetes mellitus (T2DM) and cardiovascular diseases are major public health concerns worldwide and especially in North America where 40 millions people are going to be affected by 2030 [1]. Adults with diabetes have heart disease death rates about 2 to 4 times higher than adults without diabetes [2].

It is therefore important to study T2DM risk factors. The degree of obesity, the presence of the metabolic syndrome, family history of diabetes, impaired glucose tolerance, a low physical activity level, high blood levels of triglycerides (TG), high-density lipoprotein cholesterol (HDL-c) levels under 0.91 mmol/L as well as certain ethnic groups are well-known T2DM risk factors [2]. Fish oil and omega-3 (*n*-3) polyunsaturated fatty acids (PUFA) are of particular interest because of their roles in improving the plasma lipid profile, especially by reducing plasma TG [3]. *N*-3 PUFAs represent a reasonable therapeutic strategy to improve the dyslipidemic profile in individuals with T2DM [4]. On the other hand, *n*-3 PUFA supplementation is often associated with a significant but marginal increase in fasting plasma glucose (FG) concentrations [5]. A review of 18 trials with 823 subjects on the effects of fish oil supplementation on plasma lipid levels and glycemic controls showed that the pooled weight mean difference for FG was an increase of 0.26 mmol/L after an *n*-3 PUFA supplementation with doses ranging from 3 to 18 g/d of eicosapentaenoic acid (EPA) and docosahexaenoic acid (DHA) [6].

Changes in desaturase activity are associated with insulin sensitivity, which can be estimated using the homeostasis model assessment (HOMA-IS) [7], and might be part of T2DM development. HOMA-IS and serum fatty acid composition have been associated with single-nucleotide polymorphisms (SNPs) in the fatty acid desaturase (*FADS*) gene cluster [8]. The fatty acid desaturase-1 (*FADS1*) and fatty acid desaturase-2 (*FADS2*) genes encode respectively for two desaturases: δ-5 desaturase (D5D) and δ-6 desaturase (D6D) [9]. The D5D and D6D, responsible for double bonds formation in the *n*-3 PUFA and omega-6 (*n*-6) PUFA pathways, have been associated with differences in fatty acid composition of plasma, adipose tissue, and membrane fluidity [10]. In general, D5D activity is inversely related to obesity and insulin resistance, whereas D6D activity shows positive associations [10].

Prospective studies have shown a strong inverse relationship between D5D and T2DM risk and a strong positive relation with D6D, using the desaturase indexes that estimate aggregate desaturase activity (ADA) using products to precursors ratios [11,12]. However, these studies cannot predict with certainty how confounding factors such as diet, lifestyle habits and heredity or reverse causality could explain part of the variance. It is thus relevant to study the effects of SNPs within the *FADS* gene cluster, mostly *FADS1* and *FADS2* that encode the desaturases in response to *n*-3 PUFA. By giving high doses of *n*-3 PUFA above 3 g/d, the *n*-3 PUFA pathway is promoted at the expense of the *n*-6 pathway which would potentially reduce the *n*-6:*n*-3 ratio by increasing D5D and D6D ADA and generate improvements in the metabolic profile of individuals.

The objective of the present study was to investigate whether the FG, the fasting insulin (FI), and the HOMA-IS responses to a 6-week *n*-3 PUFA supplementation were influenced by common genetic variations in the *FADS* gene cluster. We hypothesized that SNPs within the *FADS* gene cluster are associated with glycemic controls parameters after an *n*-3 PUFA supplementation.

## 2. Experimental Section

### 2.1. Study Population

A total of 254 subjects from the greater Quebec City metropolitan area were recruited between September 2009 and December 2011 through advertisements in local newspapers as well as by electronic messages sent to university students/employees. Subjects had to be aged between 18 and 50 years with a BMI between 25 and 40. Subjects were excluded from the study if they had taken *n*-3 PUFA supplements for at least 6 months prior to the study. However, only 210 subjects completed the intervention protocol and glycemic control parameters pre- and/or post-supplementation are missing for two participants [13]. Statistical analyses were then performed on 208 individuals. The experimental protocol was approved by the ethics committees of Laval University Hospital Research Center and Laval University. This trial was registered at clinicaltrials.gov as NCT01343342 [14].

### 2.2. Study Design and Diets

Subjects followed a run-in period of two weeks during which a trained registered dietitian gave individual dietary instructions. Recommendations were drawn from the *Eating Well with Canada’s Food Guide* [15]. All subjects were asked to apply these dietary recommendations and to maintain their body weight stable throughout the protocol. Among these recommendations, some specifications have been imposed to ensure the success of this study such as not to exceed two portions of fish or seafood per week (max. 150 g) and to choose, preferably, fish with white flesh and to avoid products fortified with *n*-3 PUFA during the study period. Subjects were also asked to limit their alcohol intakes to no more than two drinks per week. Subjects were not allowed to take *n*-3 PUFA supplements, including those of vegetable sources and to take vitamins or natural health products during the protocol.

After the run-in period, each participant received a bottle containing *n*-3 PUFA capsules (Ocean Nutrition, Nova Scotia, Canada) covering the following six weeks period. They had to take five capsules of fish oil per day, which gave them a total of 3–3.3 g of *n*-3 PUFA (1.9–2.2 g EPA and 1.1 g DHA) per day. Compliance was measured by bottles returning and by calculating the number of remaining capsules in the bottles at the end of the supplementation. Subjects had to report any deviations that may have occurred during the protocol. They also had to write their alcohol and fish consumption on a log sheet. Before each phase of the study, subjects received written and verbal dietary instructions by a registered dietitian.

Detailed methods were previously presented in Cormier *et al.* [13]. 

### 2.3. Anthropometric Measurements

Body weight, height, and waist girth were measured according to the procedures recommended by the Airlie Conference and were taken before the run-in period, as well as pre- and post-*n*-3 PUFA supplementation [16]. BMI was calculated as weight in kilograms divided by height in meters-squared (kg/m^2^).

### 2.4. Biochemical Parameters

Blood samples were collected from an antecubital vein into vacutainer tubes containing EDTA after 12 h overnight fast and 48 h alcohol abstinence. Blood samples were taken to identify and exclude individuals with metabolic disorders such as diabetes (FG > 7.0 mmol/L), hypertension (>140 mmHG/>90 mmHG), hypo/hyperthyroidism (based on thyroid-stimulating hormone (TSH) levels) or severe dyslipidemias [17].

Afterwards, selected participants had blood samples taken at prior and after the *n*-3 PUFA supplementation period. Plasma was separated by centrifugation (2,500 × g for 10 min at 4 °C) and samples were aliquoted and frozen for subsequent analyses. FI was measured by radioimmunoassay with polyethylene glycol separation [18]. FG concentrations were enzymatically measured [19]. HOMA-IS was calculated using the following formula: 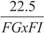 [7]. Plasma C-reactive protein (CRP) was measured by nephelometry (Prospec equipment Behring) using a sensitive assay, as described previously [20]. Plasma total cholesterol (TC) and TG concentrations were measured using enzymatic assays [21]. The high-density lipoprotein cholesterol (HDL-C) fraction was obtained after precipitation of very low-density lipoprotein and low-density lipoprotein particles in the infranatant with heparin manganese chloride [22]. Low-density lipoprotein cholesterol (LDL-C) was calculated with the Friedewald formula [23]. Apolipoprotein B-100 (ApoB100) concentrations were measured in plasma by the rocket immunoelectrophoretic method of Laurell, as previously described [24]. 

### 2.5. SNP Selection and Genotyping

SNPs in *FADS1*, *FADS2* and *FADS3* were identified using the International HapMap Project SNP database, based on the National Center for Biotechnology Information (NCBI) B36 assembly Data Rel 28. phase II + III, build 126. The *FADS* gene cluster is made of three genes that are located very close to each other among chromosome 11. Because of the head-to head orientation of *FADS1* and *FADS2* and the tail-to-tail orientation of *FADS2* and *FADS3*, we added 500 kilo-base pairs (kbp) downstream of *FADS1* and 2,500 kbp upstream of *FADS3* to cover the promoter region. Intergenic areas were also covered. Tagger procedure in Haploview V4.2 was used to determine tag SNPs (tSNPs) using a minor allele frequency (MAF) ≥3% and pairwise tagging (r^2^ ≥ 0.8). Subsequently, we examined linkage disequilibrium (LD) out of the 19 SNPs covering all common variations in the *FADS* gene cluster area, using the LD Plot procedure in Haploview V4.2. Since the majority of the SNPs within the *FADS* gene cluster are in high LD (r^2^ ≥ 0.8) with each other, 19 SNPs were sufficient to cover 96% of the entire area. The SIGMA GenElute Gel Extraction Kit (Sigma-Aldrich Co., St. Louis, MO, USA) has been used to extract genomic DNA. Selected SNPs of the *FADS* gene cluster (rs174456, rs174627, rs482548, rs2072114, rs12807005, rs174448, rs2845573, rs7394871, rs7942717, rs74823126, rs174602, rs498793, rs7935946, rs174546, rs174570, rs174579, rs174611, rs174616 and rs968567) have been genotyped using validated primers and TaqMan probes (Life Technologies Corporation, Carlsbad, CA, USA). DNA was mixed with TaqMan Universal PCR Master Mix (Life Technologies Corporation, Carlsbad, CA, USA), with a gene-specific primer and with probe mixture (predeveloped TaqMan SNP Genotyping Assays; Life Technologies Corporation, Carlsbad, CA, USA) in a final volume of 10 μL. Genotypes were determined using a 7500 RT-PCR System and analyzed using ABI Prism SDS version 2.0.5 (Life Technologies Corporation, Carlsbad, CA, USA). The Exonic Splicing Enhancer (ESE) finder Webbased program was used to determine the potential effect of variants of the *FADS* gene cluster on pre-mRNA splicing. All tests were run under default threshold values [25].

### 2.6. Statistical Analyses

All genotype distributions were tested for any deviation from Hardy-Weinberg equilibrium (HWE) using the ALLELE procedure in SAS Genetics v9.3 (SAS Institute Inc., Cary, NC, USA). All SNPs were in HWE except one: rs7935946. Significance testing for LD coefficient D was obtained using a chi-square test, likelihood ratio and Fisher exact test (*p* ≤ 0.01).

All other statistical analyses were carried out using SAS v9.3 (SAS Institute Inc., Cary, NC, USA). Normal distribution of outcome variables was evaluated looking at the box-plot, and also skewness and kurtosis ranges for normal distribution. When needed, outcome variables non-normally distributed were log10-transformed. A linear regression using the stepwise bidirectional elimination approach was used to assess which SNPs could explain part of the glycemic control parameters’ variance where the effects of the 18 SNPs that were in HWE and the effects of age, sex, and BMI were included in the statistical model. The MIXED procedure was used to test for the effects of the genotype, the supplementation and the genotype by supplementation interaction for each SNP on FG, FI and HOMA-IS levels when age, sex and BMI were included in the model. The repeated statement was used to indicate the within subjects (repeated) variables. Genotypic groups were assessed as three groups expressed as Major Allele Homozygotes (11), Heterozygotes (12) and Minor Allele Homozygotes (22). For some SNPs, Heterozygotes and Minor Allele Homozygotes were grouped if the genotypic frequency of the Minor Allele Homozygotes was under 5%. Since polymorphisms tested in complex diseases rarely account for a large amount of variance, characterized by very low *p*-values (*p* < 0.001), we decided to present the results without correction for multiple testing and using a *p*-value ≤0.05. Statistical significance was defined as *p* ≤ 0.05. 

## 3. Results and Discussion

Baseline characteristics of study participants are presented in Table 1. Since we recruited overweight participants, the mean BMI is above 25 kg/m^2^ in both men and women. Gender differences are evident with respect to weight, TC/HDL-C ratio, HDL-C, TG and CRP levels. In both men and women, the *n*-3 PUFA supplementation was associated with a decrease of plasma TG levels as well as with an increase of FG levels [5].

**Table 1:** Baseline characteristics of the study sample before *n-3* polyunsaturated fatty acids (PUFA) supplementation.

	All ^1^	Men ^1^	Women ^1^
Population. Men/Women	208	96 (46.2%)	112 (53.8%)
Age (years)	30.8 ± 8.7	31.2 ± 8.1	30.5 ± 9.1
Weight (kg) ^3^	81.4 ± 13.9	87.2 ± 13.4	76.4 ± 12.3
BMI (kg/m^2^) ^2,3^	27.8 ± 3.7	27.5 ± 3.6	28.2 ± 3.8
Waist circumference (cm) ^3^	93.3 ± 10.8	94.8 ± 11.0	92.0 ± 10.4
Cholesterol (mM) ^4^			
Total	4.82 ± 1.00	4.80 ± 1.00	4.83 ± 1.02
HDL	1.46 ± 0.39	1.29 ± 0.31	1.61 ± 0.39
LDL	2.79 ± 0.87	2.91 ± 0.87	2.69 ± 0.86
Total chol./HDL ratio ^4^	3.49 ± 1.04	3.91 ± 1.13	3.12 ± 0.80
Triacylglycerols (mM) ^2,4^	1.23 ± 0.64	1.32 ± 0.74	1.15 ± 0.53
ApoB100 (g/L) ^4^	0.86 ± 0.25	0.89 ± 0.25	0.84 ± 0.25
CRP (mg/L) ^2,4^	3.13 ± 7.10	1.66 ± 2.45	4.39 ± 9.24
Glycemic controls			
Glucose (mM) ^4^	4.95 ± 0.52	5.09 ± 0.44	4.83 ± 0.56
Insulin (ρ/L) ^4^	82.51 ± 35.61	79.50 ± 32.19	85.04 ± 38.20

^1^ Values are means ± SD. ^2^ *p*-value derived from log_10_-transformed. ^3^ Results were adjusted for age. ^4^ Results were adjusted for age and BMI.

A previous paper from our group reported that *n*-3 PUFA supplementation slightly increased FG in the present study (mean ± SD, pre-*n*-3 PUFA: 4.95 ± 0.46; post-*n*-3 PUFA: 5.06 ± 0.49, *p* = 0.02) [5]. We then wanted to test whether SNPs of the *FADS* gene cluster may modulate glycemic control parameters’ response after the 6-week supplementation period. 

All SNPs were in HWE except one: rs7935946. This SNP was not considered for further analysis. Therefore, associations with 18 SNPs were tested in statistical analyses. The percentage of gene coverage with these 18 SNPs was of 90%. 

To validate the presence of associations between glycemic control parameters and SNPs of the *FADS* gene cluster, all SNPs were included in a linear regression model with values post-*n*-3 PUFA supplementation as the dependent variable, adjusted for baseline values (FG, FI or HOMA-IS levels), age, sex and BMI. With the use of the stepwise bidirectional selection method, 2 SNPs were associated with FG levels: rs482548 (2.05%, *p* = 0.01) and rs498793 (1.75%, *p* = 0.02); 1 SNP was associated with FI levels: rs174602 (0.89%, *p* = 0.04); and 2 SNPs were associated with HOMA-IS: rs174602 (1.97%, *p* = 0.005) and rs482548 (0.90% *p* = 0.05) (Data not shown).

To test the potential interaction between the *FADS* gene polymorphisms and the *n*-3 PUFA supplementation on plasma FG, FI and HOMA-IS levels, a general linear model adjusted for sex, age and BMI was used in order to verify whether the genotype, the supplementation or the interaction (genotype by supplementation) were associated with plasma levels of glycemic control parameters. As shown in Table 2, independently of the genotype, the supplementation was associated with FG concentrations (significant associations with 16 SNPs of the *FADS* gene cluster) meaning that the supplementation had an independent effect on plasma FG levels, as expected. One SNP (rs482548) was associated with plasma FG levels meaning that the genotype for that particular polymorphism may modulate the plasma FG during the intervention. A significant genotype by supplementation interaction effect on FG levels was observed for rs482548 (*p* = 0.008). For FI levels, a genotype effect was observed with one SNP (rs174456). For HOMA-IS, several genotype by supplementation interaction effects were observed for rs7394871, rs174602, rs174570, rs7482316 and rs482548 (*p* = 0.03, *p* = 0.01, *p* = 0.03, *p* = 0.05 and *p* = 0.07 respectively) (Table 2). Table 3 shows the genotype by supplementation interaction effect on HOMA-IS and FG levels by genotypic groups and the β-values for the four significant SNPs after the 6-week *n*-3 PUFA supplementation.

**Table 2:** Effect of the genotype, the *n-3* PUFA supplementation and the interaction (genotype by supplementation) on fasting glucose (FG), fasting insulin (FI) and homeostasis model assessment of insulin sensitivity (HOMA-IS) levels (*n* = 208).

	FG				FI ^1^			HOMA-IS		
	Genotype	Suppl.	Interaction		Genotype	Suppl.	Interaction	Genotype	Suppl.	Interaction
rs174456	0.28	0.06	0.42		0.04	0.14	0.16	0.84	0.77	0.84
rs174627	0.81	0.002	0.93		0.98	0.97	0.74	0.89	0.52	0.90
rs482548	0.05	<0.0001	0.008		0.18	0.59	0.16	0.11	0.13	0.07
rs2072114	0.07	0.05	0.11		0.25	0.36	0.23	0.65	0.49	0.71
rs12807005	0.66	0.04	0.38		0.30	0.41	0.33	0.90	0.83	0.99
rs174448	0.79	0.002	0.65		0.85	0.66	0.84	0.61	0.27	0.45
rs2845573	0.35	0.03	0.55		0.67	0.81	0.92	0.41	0.19	0.24
rs7394871	0.58	0.05	0.44		0.41	0.55	0.29	0.05	0.04	0.03
rs7942717	0.93	0.004	0.83		0.27	0.67	0.67	0.99	0.40	0.61
rs7482316	0.48	0.003	0.83		0.05	0.39	0.08	0.03	0.09	0.05
rs174602	0.88	0.0006	0.79		0.46	0.72	0.11	0.09	0.12	0.01
rs498793	0.60	<0.0001	0.16		0.50	0.84	0.63	0.26	0.33	0.25
rs174546	0.34	0.03	0.52		0.44	0.58	0.74	0.42	0.28	0.42
rs174570	0.67	0.004	0.80		0.33	0.61	0.18	0.07	0.08	0.03
rs174579	0.58	0.0004	0.77		0.97	0.88	0.71	0.38	0.39	0.35
rs174611	0.88	0.004	0.73		0.75	0.86	0.82	0.54	0.44	0.65
rs174616	0.49	0.0003	0.27		0.44	0.45	0.35	0.21	0.61	0.11
rs968567	0.74	0.0007	0.84		0.51	0.71	0.68	0.68	0.45	0.71

The MIXED procedure implemented in SAS version 9.3 was used to test interaction effects. All results were adjusted for BMI, age and sex. ^1^ *p*-values derived from log_10_-transformed for insulin levels.

**Table 3:** Genotype by supplementation interaction effect on HOMA-IS and FG levels after a 6-week *n*-3 PUFA supplementation.

	Pre-*n*-3 PUFA		Post-*n*-3 PUFA		P ^1^		β-values ± SE
	11	12 + 22	11	12 + 22			
**HOMA-IS**	0.067 ± 0.025	0.063 ± 0.027	0.068 ± 0.030	0.056 ± 0.022	0.01	**11**	0.4169 ± 0.0375
rs174602						**12 + 22**	0.4391 ± 0.0379
**HOMA-IS**	0.066 ± 0.025	0.065 ± 0.029	0.066 ± 0.029	0.057 ± 0.025	0.03	**11**	0.4106 ± 0.0378
rs174570						**12 + 22**	0.4248 ± 0.0384
**HOMA-IS**	0.065 ± 0.025	0.068 ± 0.033	0.065 ± 0.028	0.059 ± 0.025	0.03	**11**	0.4150 ± 0.0378
rs7394871						**12 + 22**	0.4319 ± 0.0391
**FG**	4.96 ± 0.43	4.92 ± 0.54	5.02 ± 0.48	5.15 ± 0.52	0.008	**11**	1.5753 ± 0.7327
rs482548						**12 + 22**	1.2278 ± 0.7443

11 stands for major allele homozygotes and 12 + 22 stands for heterozygotes and minor allele homozygotes grouped together. The MIXED procedure implemented in SAS version 9.3 was used to test interaction effects. All results were adjusted for BMI, age and sex. ^1^ *p*-values for the interaction (genotype by supplementation).

**Figure 1:**
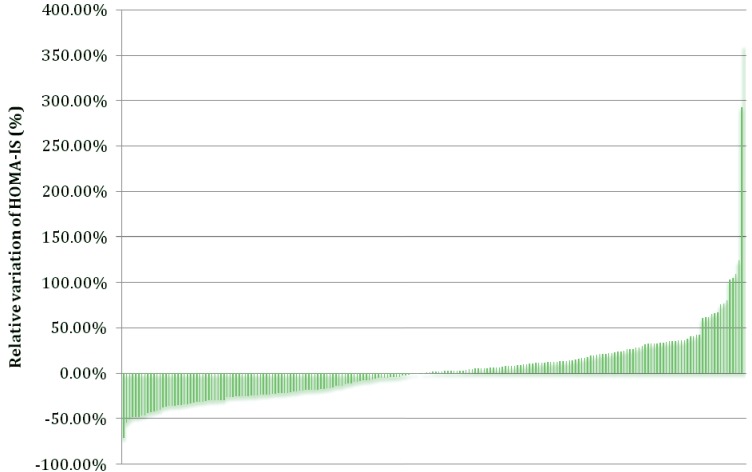
Inter-individual variability of HOMA-IS after a 6-week *n*-3 PUFA supplementation (*n* = 208).

Insulin resistance is a condition in which glucose intakes by sensitive tissues decreased. Therefore, insulin resistance and insulin sensitivity are strong predictors of T2DM [26]. Individuals affected with T2DM also have higher cardiovascular risk. An *n*-3 PUFA supplementation may exert beneficial effects on cardiovascular risk on that specific population. Connor *et al.* showed that *n*-3 PUFA intake (6 g/d of EPA + DHA for 6 months), along with oral therapy for T2DM, can lower TG concentrations, with no adverse effects on glycemic control [27]. In addition to decrease TG levels, an *n*-3 PUFA supplementation may increase slightly FG levels. As previously reported by our research group, FG increased by 2.44% ± 49.55% (*p* = 0.0004) after the 6-week *n*-3 PUFA supplementation [5]. The high standard deviation associated with changes in FG levels shows that there is a large inter-individual variability in FG response following an *n*-3 PUFA supplementation. Because FG is part of the HOMA-IS formula and correlates negatively with this model, it is believed that the high inter-individual variability may also be seen in HOMA-IS values. As shown in Figure 1, 99 individuals decreased their HOMA-IS (mean ± SD; −23.2 ± 14.3%) while 107 individuals increased their HOMA-IS (mean ± SD; 30.4 ± 48.4%) after the *n*-3 PUFA supplementation.

The fatty acid profile of membrane phospholipids and the activity of desaturases are strongly linked to the incidence of T2DM [11]. The large inter-individual variability in HOMA-IS response after an *n*-3 PUFA supplementation could be due to different dietary factors such as a diet rich in saturated and trans fats, a reduced enzymatic activity of D5D and D6D, a low dietary contribution of C20-C22 PUFAs or high amount of 18:2 *n*-6 (linoleic acid) in the diet [28]. Increasing the content of longer chain PUFAs increases membrane fluidity, the number of insulin receptors and the action of insulin [29,30]. Remarkably, it has been demonstrated that an increase in cell membrane fluidity increases both the number of insulin receptors on the membrane and their affinity to insulin, thus improving insulin sensitivity [31,32]. Defects in D5D and D6D could induce decreases in C20-C22 PUFAs in skeletal muscle membrane phospholipids that lead to less unsaturation of membrane phospholipids. Less unsaturation of membrane phospholipids could possibly affect glucose metabolism. 

The mechanism by which insulin resistance increases cardiovascular risk is unclear. However, the emerging literature demonstrates that genetic factors may be involved [33,34]. D5D and D6D, key enzymes in the PUFAs metabolism and encoded by genes *FADS1* and *FADS2*, may influence glucose metabolism [35]. The D6D activity may be strongly and positively related to the incidence of T2DM, whereas the literature reports an inverse relationship with the activity of D5D [36]. These desaturases have a concomitant activity in the *n*-3 and *n*-6 PUFA metabolism, creating a competition where the *n*-6 PUFA pathway is often favored [37]. An unbalanced ratio of *n*-3:*n*-6 due to the enzymatic activity of desaturases involved in PUFA metabolism might alter glucose metabolism [34].

A recent study published by Manning *et al.* has identified many loci implicated in T2DM, including SNPs in *FADS1*, which were associated with FG [38]. In the present study, the majority of SNPs that showed associations with glycemic control parameters were from the *FADS2* gene, but as the study covered 90% of all common genetic variations of the *FADS* gene cluster, some SNPs may be in LD with other SNPs found on *FADS1* or *FADS3* that may be potentially functional (Table 4).

**Table 4:** Description of the selected polymorphisms in the fatty acid desaturase (*FADS*) gene cluster.

Gene	dbSNP No. ^1^	Sequence ^2^	Position	MAF	Genotype/Frequency		
					11	12	22
*FADS3*	rs174456	CTAC[A/C]TGGC	Intron	0.299	A/A (*n* = 102)	A/C (*n* = 89)	C/C (*n* = 18)
					0.488	0.426	0.086
Intergenic *FADS2*-*FADS3*	rs174627	TCTG[C/T]GTAG	Intergenic	0.124	G/G (*n* = 159)	A/G (*n* = 48)	A/A (*n* = 2)
					0.761	0.230	0.010
*FADS2*	rs482548	ACAC[C/T]GTGG	3' UTR	0.126	C/C (*n* = 161)	C/T (*n* = 40)	T/T (*n* = 6)
					0.778	0.193	0.029
*FADS2*	rs2072114	GTTC[A/G]GGTC	Intron	0.110	A/A (*n* = 167)	A/G (*n* = 38)	G/G (*n* = 4)
					0.799	0.182	0.019
Intergenic *FADS1*-*FADS2*	rs12807005	CATG[A/G]ATCA	Intergenic	0.012	G/G (*n* = 204)	A/G (*n* = 5)	A/A (*n* = 0)
					0.976	0.024	0.000
Intergenic *FADS2*-*FADS3*	rs174448	CTGA[C/T]TTCT	Intergenic	0.363	A/A (*n* = 78)	A/G (*n* = 109)	G/G (*n* = 21)
					0.375	0.524	0.101
*FADS2*	rs2845573	CTCA[C/T]GTTA	Intron	0.081	A/A (*n* = 177)	A/G (*n* = 30)	G/G (*n* = 2)
					0.847	0.144	0.010
*FADS3*	rs7394871	GGAC[A/C]CCTG	Intron	0.072	C/C (*n* = 181)	A/C (*n* = 26)	A/A (*n* = 2)
					0.866	0.124	0.010
*FADS3*	rs7942717	AACG[A/G]GTGC	Intron	0.117	A/A (*n* = 161)	A/G (*n* = 47)	G/G (*n* = 1)
					0.770	0.225	0.005
Intergenic *FADS2*-*FADS3*	rs7482316	TCAA[A/G]CTGC	Intergenic	0.103	A/A (*n* = 168)	A/G (*n* = 39)	G/G (*n* = 2)
					0.804	0.187	0.010
*FADS2*	rs174602	ACCC[A/G]TCCT	Intron	0.184	T/T (*n* = 141)	C/T (*n* = 59)	C/C (*n* = 9)
					0.675	0.282	0.043
*FADS2*	rs498793	TAAC[A/G]CAGG	Intron	0.456	C/C (*n* = 62)	C/T (*n* = 99)	T/T (*n* = 43)
					0.098	0.717	0.186
*FADS2*	rs7935946	GTTC[C/T]GGGA	Intron	0.041	C/C (*n* = 195)	C/T (*n* = 11)	T/T (*n* = 3)
					0.933	0.053	0.014
*FADS1*	rs174546	CTGC[C/T]TTGG	3' UTR	0.297	C/C (*n* = 103)	C/T (*n* = 86)	T/T (*n* = 19)
					0.498	0.412	0.091
*FADS2*	rs174570	TTGA[C/T]GTAG	Intron	0.125	C/C (*n* = 159)	C/T (*n* = 46)	T/T (*n* = 3)
					0.764	0.221	0.014
*FADS2*	rs174579	CTTT[C/T]CAGG	Intron	0.202	C/C (*n* = 127)	C/T (*n* = 78)	T/T (*n* = 3)
					0.611	0.375	0.014
*FADS2*	rs174611	TGGA[C/T]CCTG	Intron	0.258	T/T (*n* = 113)	C/T (*n* = 84)	C/C (*n* = 12)
					0.541	0.402	0.057
*FADS2*	rs174616	CTCA[C/T]GTTC	Intron	0.498	A/A (*n* = 51)	A/G (*n* = 108)	G/G (*n* = 50)
					0.244	0.517	0.239
*FADS2*	rs968567	CCGG[A/G]AGCT	5' UTR	0.160	G/G (*n* = 144)	A/G (*n* = 63)	A/A (*n* = 2)
					0.689	0.301	0.010

**^1^** dbSNP No. from HapMap Data Rel 28 Phase II + III, August 10 on NCBI b36 Assembly dbSNP b126 database; **^2^** Genes sequences from dbSNP short genetics variations NCBI reference assembly.

A study by Brenner has shown that insulin influences the activity of D5D and D6D [35]. Accordingly to these results, changes in ADA might be induced by aberrant insulin concentrations in prediabetic states [35]. Kim *et al.* have shown in a study on Korean men in 2011 that SNPs rs174575 (in high LD with rs174579, r^2^ = 0.864) and rs2727270 (in high LD with rs2072114, r^2^ = 1.0) were associated with insulin resistance [8]. In the present study, only rs174456 was associated with FI levels. Differences may exist in allele frequencies, environment or the absence of common SNPs between both populations from two different ethnic groups potentially explaining these different SNP association pattern [39].

Many studies have detected a significant relation of the genotype rs174547 (in high LD with rs174546, r^2^ = 1.0) with FG levels [40,41]. Overall, in the present study, rs174546 was not associated with FG, but was associated with TG levels pre-*n*-3 PUFA supplementation (*p* = 0.002) and a trend was also observed for TG levels post-*n*-3 PUFA supplementation (*p* = 0.07) [13]. Out of the 18 SNPs studied, only rs482548 was associated with FG levels after *n*-3 supplementation.

Regarding the possible effect of variants of rs482548, analysis of the scores obtained with ESE revealed that no variants markedly alter the binding capacity of putative ESE elements, either by reducing their values or by creating new binding site for SR proteins.

A key strength of the study is that it was an interventional study design and that high doses of *n*-3 were administered to participants. Additionally, it would have been interesting to use an oral glucose tolerance test (OGTT) and to measure insulin secretion by dosing C-peptide. The actual study does not allow to go further in the analyses of glycemic control parameters, suggesting that more studies are needed to better understand the impact of the *FADS* gene cluster polymorphisms on T2DM risk. Also, the short run-in period (2 weeks) may not be sufficient to take into account high basal levels of *n*-3 PUFAs in phospholipids membranes. Compliance was assessed from the return of containers and capsules (87%) to determine the quantity consumed and by measuring membrane phospholipid composition. 

## 4. Conclusions

In conclusion, the importance of desaturases, which are the key enzymes in the PUFAs metabolism in the development of T2DM, deserves special attention. Our results show that several SNPs involved in the *FADS* gene cluster are associated with glycemic control parameters, mainly with HOMA-IS, in response to an intervention with high doses of *n*-3 PUFA. These SNPs are of particular interest because of their potential effects on glucose metabolism. Dietary *n*-3 supplements could be used as add-ons to a well-balanced diet and can be part of a healthy lifestyle in treating T2DM associated dyslipidemia.
